# Molecular Basis for Strain Variation in the *Saccharomyces cerevisiae* Adhesin Flo11p

**DOI:** 10.1128/mSphere.00129-16

**Published:** 2016-08-17

**Authors:** Subit Barua, Li Li, Peter N. Lipke, Anne M. Dranginis

**Affiliations:** a Department of Biological Sciences, St. John’s University, Queens, New York, USA; b Biology Department, Brooklyn College of City University of New York, Brooklyn, New York, USA; c Biology Program, The Graduate Center of City University of New York, New York, New York, USA; University of Texas Health Science Center

**Keywords:** FLO11, adhesion domain, homotypic binding, phenotype variation, sequence variation

## Abstract

*FLO11* encodes a yeast cell wall flocculin that mediates a variety of adhesive phenotypes in *Saccharomyces cerevisiae*. Flo11p is implicated in many developmental processes, including flocculation, formation of pseudohyphae, agar invasion, and formation of microbial mats and biofilms. However, Flo11p mediates different processes in different yeast strains. To investigate the mechanisms by which *FLO11* determines these differences in colony morphology, flocculation, and invasion, we studied gene structure, function, and expression levels. Nonflocculent *Saccharomyces cerevisiae* Σ1278b cells exhibited significantly higher *FLO11* mRNA expression, especially in the stationary phase, than highly flocculent *S. cerevisiae* var. *diastaticus*. The two strains varied in cell surface hydrophobicity, and Flo11p contributed significantly to surface hydrophobicity in *S. cerevisiae* var. *diastaticus* but not in strain Σ1278b. Sequencing of the *FLO11* gene in *S. cerevisiae* var. *diastaticus* revealed strain-specific differences, including a 15-amino-acid insertion in the adhesion domain. Flo11p adhesion domains from strain Σ1278b and *S. cerevisiae* var. *diastaticus* were expressed and used to coat magnetic beads. The adhesion domain from each strain bound preferentially to homologous cells, and the preferences were independent of the cells in which the adhesion domains were produced. These results are consistent with the idea that strain-specific variations in the amino acid sequences in the adhesion domains cause different Flo11p flocculation activities. The results also imply that strain-specific differences in expression levels, posttranslational modifications, and allelic differences outside the adhesion domains have little effect on flocculation.

**IMPORTANCE** As a nonmotile organism, *Saccharomyces cerevisiae* employs the cell surface flocculin Flo11/Muc1 as an important means of adapting to environmental change. However, there is a great deal of strain variation in the expression of Flo11-dependent phenotypes, including flocculation. In this study, we investigated the molecular basis of this strain-specific phenotypic variability. Our data indicate that strain-specific differences in the level of flocculation result from significant sequence differences in the *FLO11* alleles and do not depend on quantitative differences in *FLO11* expression or on surface hydrophobicity. We further have shown that beads coated with amino-terminal domain peptide bind preferentially to homologous cells. These data show that variability in the structure of the Flo11 adhesion domain may thus be an important determinant of membership in microbial communities and hence may drive selection and evolution.

## Abstract (precis)

As a nonmotile organism, *Saccharomyces cerevisiae* employs the cell surface flocculin Flo11/Muc1 as an important means of adapting to environmental change. However, there is a great deal of strain variation in the expression of Flo11-dependent phenotypes, including flocculation. In this study, we investigated the molecular basis of this strain-specific phenotypic variability. Our data indicate that strain-specific differences in the level of flocculation result from significant sequence differences in the *FLO11* alleles and do not depend on quantitative differences in *FLO11* expression or on surface hydrophobicity. We further have shown that beads coated with amino-terminal domain peptide bind preferentially to homologous cells. These data show that variability in the structure of the Flo11 adhesion domain may thus be an important determinant of membership in microbial communities and hence may drive selection and evolution.

## INTRODUCTION

In the brewing industry, cell-cell adhesion is economically important because yeast cells spontaneously adhere in clumps in a process call flocculation. This adherence facilitates removal from the medium by sedimentation at the end of fermentation. In general, flocculation in *Saccharomyces cerevisiae* is described as reversible calcium-dependent asexual cell-cell aggregation (1). Flocculation can result from the expression of adhesins from either of two gene families: *FLO11* (formerly also called *MUC1*) or any of several members of the *FLO1* family. The *FLO1*-like flocculins have N-terminal regions with the structure and function of C-type lectins specific for alpha-mannosides or for both alpha-glucosides and alpha-mannosides. *FLO1*-like flocculins are often expressed at the end of fermentation in beer strains (1–3). In contrast, *FLO11-*dependent flocculation has been observed in wine-fermenting strains, including those that make sherry, where Flo11p mediates the formation of a hydrophobic yeast “flor” that grows at the air-liquid interface (4, 5). A recent structural study showed that the N-terminal region of Flo11p from strain S288C forms a unique structure similar to fibronectin type III repeats. This domain mediates homotypic binding through hydrophobic-effect interactions and has no demonstrable lectin activity (6, 7). Various factors, such as cell density, surface charge, and pH, and environmental factors, such as oxygen limitation, nutrient limitation, and cell surface hydrophobicity, influence the expression and flocculation activity of Flo11p (8–11).

In *S. cerevisiae*, *FLO11-*mediated cell-cell adhesion and cell-substrate adhesion are required for many developmental processes and for the formation of multicellular structures. Depending on the specific strain of *S. cerevisiae*, Flo11p is required for the formation of flocs and pseudohyphae, agar invasion, adherence to plastic, and biofilm development (12–15). The different *FLO11*-dependent phenotypes of two strains of *S. cerevisiae* used in this study are summarized in Table 1 (16). For example, expression of *FLO11* is necessary for flocculation in *S. cerevisiae* var. *diastaticus* (16). On the other hand, yeast cells of the strain Σ1278b background do not flocculate, although they do express *FLO11* (17). On solid media, limitation of utilizable nitrogen compounds induces strain Σ1278b diploid cells to form pseudohyphae composed of polarized invasive filaments that form chains of elongated cells. On rich medium, lack of glucose triggers the penetration into agar of filaments of haploid but not diploid cells; this process is known as invasive growth (12–15). In strain Σ1278b, both haploid invasive growth and diploid pseudohypha formation are *FLO11* dependent (14). Moreover, yeast cells of the strain Σ1278b background have been shown to exhibit a characteristic morphology on mat formation in the *FLO11*-dependent manner. On the other hand, yeast cells of the *S. cerevisiae* var. *diastaticus* background express Flo11p but have a reduced ability to invade or form pseudohyphae. *S. cerevisiae* var. *diastaticus* does not form mats (18, 19). Interestingly, both strain Σ1278b and *S. cerevisiae* var. *diastaticus* can adhere to a plastic surface when grown in low-glucose medium and remain attached after repeated washes; this activity is also dependent on the expression of *FLO11* (16).

**TABLE 1:** Phenotypic variation in the *S. cerevisiae* var. *diastaticus* and strain Σ1278b backgrounds

Phenotype	Variation^a^ **in strain background:**	
	*S. cerevisiae* var. *diastaticus*	Σ1278b
Flocculation	+++	−
Agar invasion	−	+++
Pseudohyphae	−	+++
Cell adherence to plastic	+++	++
Mat structure formation	−	Central hub, channels, rims
Colony morphology	Smooth	Wrinkled, lacy structure

a Plus signs represent the degrees of cell adhesion phenotypes, with +++ being the maximum, and a minus sign indicates that the phenotype is not observed (19).

Using an *in vitro* bead binding assay, we have shown that purified Flo11p from *S. cerevisiae* var. *diastaticus* exhibits homotypic adhesion properties (16). We report here on the sequence, gene expression, and flocculation properties of Flo11p from *S. cerevisiae* var. *diastaticus* and strain Σ1278b. The results demonstrate the sequence specificity of Flo11p-dependent phenotypic variations.

## RESULTS

### *FLO11* mRNA expression is highest in stationary phase.

**FIG 1:**
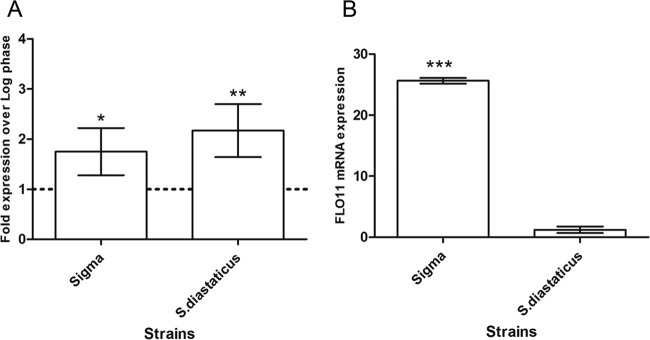
qRT-PCR expression of *FLO11* transcripts. Samples (*n* = 6) were taken during the logarithmic growth phase (*A*_600_ of ~0.8) and the stationary phase (*A*_600_ of ~2.8). The relative expression of *FLO11* in each strain was normalized to the relative expression of the housekeeping gene *PDA1*. (A) Graph representing the expression of *FLO11* transcripts by each strain in the stationary phase compared to that in the log phase. (B) Graph representing a comparison of the *FLO11* transcripts of strain Σ1278b (Sigma) and *S. cerevisiae* var. *diastaticus* in the stationary phase. Unpaired *t* tests were used, and data are expressed as the mean ± the standard deviation; asterisks denote statistically significant differences (*, *P* < 0.05; **, *P* < 0.01; ***, *P* < 0.001).

We compared *FLO11* expression levels in exponential- and stationary-phase cells of *S. cerevisiae* strain Σ1278b and *S. cerevisiae* var. *diastaticus*. Quantitative reverse transcription (qRT)-PCR analysis of *FLO11* transcripts showed that the expression of *FLO11* mRNA was significantly higher in both strains in the stationary phase than in the log phase (Fig. 1A). The *FLO11* mRNA expression level of *S. cerevisiae* var. *diastaticus* was ~2.1-fold higher in the stationary phase than in the log phase, and that of strain Σ1278b was ~1.75-fold higher. Moreover, in stationary-phase cells, *FLO11* mRNA expression was 21-fold higher in strain Σ1278b than in *S. cerevisiae* var. *diastaticus* (Fig. 1B).

### Contribution of FLO11p to surface hydrophobicity.

**FIG 2:**
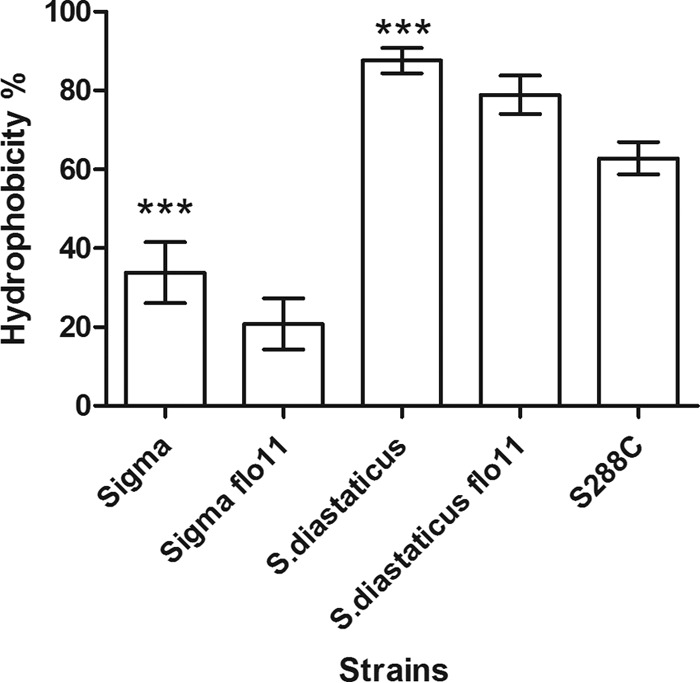
Water-hexane partitioning of wild-type and *flo11*Δ disruptant strains. Percent hydrophobicity indicates the fraction of cells partitioning in the hexane layer. Unpaired *t* tests were used, and data are expressed as the mean ± the standard deviation (*n* = 14 per strain); asterisks denote statistically significant differences from the corresponding deletion strain (***, *P* < 0.001).

Surface hydrophobicity, as measured by the octane/aqueous partition coefficient, varied between strains in the stationary phase. *S. cerevisiae* var. *diastaticus* was highly hydrophobic (87% in the organic phase), whereas only 33% of strain Σ1278b cells partitioned to the organic phase (Fig. 2). In the corresponding *flo11* deletion strains, there was a reduction in hydrophobicity. The *S. cerevisiae* var. *diastaticus* flo11Δ mutant showed a 9% reduction to 78% in the organic phase, and in the strain Σ1278b *flo11Δ* mutant, the organic phase contained only 20% of the cells. In contrast, in *S. cerevisiae* S288C, 62% of the cells partitioned to the organic phase. These results indicate that Flo11p contributed a minor fraction of the surface hydrophobicity of *S. cerevisiae* var. *diastaticus* and about 40% of that of strain Σ1278b, consistent with the strain’s intrinsically lower hydrophobicity and greater *FLO11* expression.

### Comparison of Flo11p sequences of nonflocculent strain Σ1278b and highly flocculent *S. cerevisiae* var. *diastaticus*.

To determine whether the strain-specific phenotypes are a consequence of primary sequence differences in *FLO11*, we sequenced the gene of highly flocculent *S. cerevisiae* var. *diastaticus* and compared the deduced amino acid sequence with those from strains S288C (20) and Σ1278b (21). We defined six regions of Flo11p: the secretion signal sequence (amino acids 1 to 21); the adhesion domain (AD; amino acids 22 to 209); three types of Ser- and Thr-rich repeat sequences, RI (amino acids 210 to 250), RII (amino acids 312 to 876), and RIII (amino acids 930 to 1086); and a Cys-rich C-terminal region including a signal sequence for the addition of a glycosyl phosphatidylinositol (GPI) anchor.

**FIG 3:**
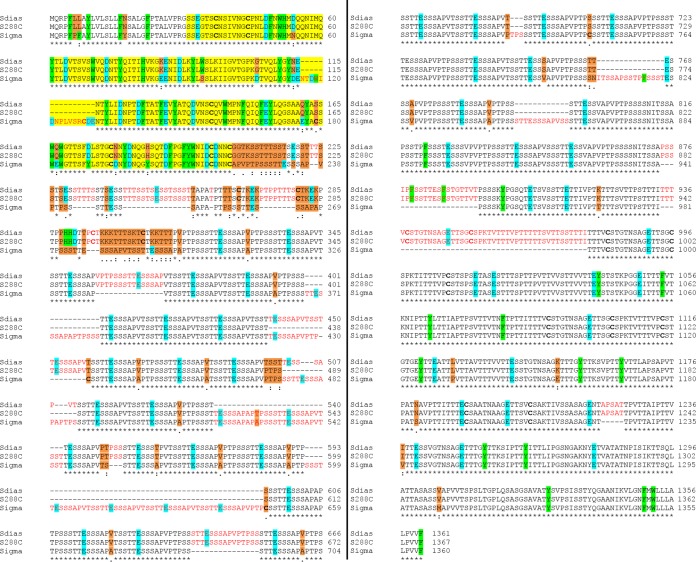
Alignment of multiple Flo11p sequences. The Flo11p sequences of *S. cerevisiae* var. *diastaticus* and strains S288C and Σ1278b were aligned with Clustal Omega. The Flo11p N-terminal AD is highlighted in yellow, and insertions in the sequences are highlighted in red. The single amino acid differences among *S. cerevisiae* var. *diastaticus* and strains S288C and Σ1278b are orange. Relevant aromatic residues are light green, and acidic residues are turquoise. Cysteine residues are in bold black. Asterisks indicate positions with identical amino acids, colons indicate positions with highly similar amino acids, and periods indicate positions with similar amino acids.

Overall, *S. cerevisiae* var. *diastaticus* Flo11p (1,361 amino acids) was highly similar to strain S288C (1,367 amino acids) (Fig. 3). All of the differences between these two strains were in the RII region. There were four amino acid substitutions at positions 498 to 501 and three insertions/deletions (insertions at positions 439 to 450 and 502 to 510 of *S. cerevisiae* var. *diastaticus* and an insertion at positions 520 to 546 of strain S288C).

In contrast, between *S. cerevisiae* var. *diastaticus* and strain Σ1278b, there were numerous substitutions, as well as 20 insertion/deletion events, across all of the regions of Flo11p. (In comparison to the published *FLO11* sequence of strain Σ1278b [21], the *FLO11* sequence of strain Σ1278b-MIT is identical in the AD, RI, and RIII regions; the RII region is 150 residues shorter.) Interestingly, nonflocculent strain Σ1278b has a 15-amino-acid insertion in the AD at positions 115 to 130 (NTDWIDNPLVSRCDE), including four acidic residues (Fig. 3, red font in the yellow AD). There are also substitutions in the AD, with seven of these point mutations, K85E, G106D, N113D, Q176E, Q182E, and N195D, and H2O2Y, making the region more acidic. These are offset by D54N, D189Y, and an added Arg in the insertion. There are two changes involving aromatic residues, W94S and H2O2Y (Fig. 3, light green). In addition, there are two more Cys residues in strain Σ1278b, one in the insert and the other a substitution at position S179C.

The repeat region of nonflocculent strain Σ1278b Flo11p also differs from that of highly flocculent *S. cerevisiae* var. *diastaticus*. There are numerous insertion/deletion events in the tandem repeats. This finding is consistent with a study showing that the number of tandem repeats is variable (22). For example, while five identical copies of a five-amino-acid (STTTS) repeat in RI and RIII at positions 211 to 215, 221 to 225, 231 to 235, 241 to 245, and 1018 to 1022 in *S. cerevisiae* var. *diastaticus* and at positions 211 to 215, 221 to 225, 231 to 235, 241 to 245, and 1024 to 1028 in strain S288C Flo11p are highly conserved, in strain Σ1278b, there is only one copy, at positions 1022 to 1026 (Fig. 3). In addition, in strain Σ1278b, there are nine identical copies of a 15-amino-acid motif (VPTPSSSTTESSSAP) in RII at positions 223 to 237, 238 to 252, 469 to 483, 685 to 699, 727 to 741, 757 to 771, 772 to 786, 829 to 843, and 901 to 915; in contrast, there are 17 copies in *S. cerevisiae* var. *diastaticus* at positions 314 to 328, 329 to 343, 356 to 370, 395 to 409, 470 to 484, 533 to 547, 575 to 589, 590 to 604, 632 to 646, 647 to 661, 662 to 676, 701 to 715, 716 to 730, 731 to 745, 758 to 772, 773 to 787, and 833 to 847 and 18 copies in strain S288C at positions 314 to 328, 329 to 343, 356 to 370, 395 to 409, 458 to 472, 485 to 499, 512 to 526, 581 to 595, 596 to 610, 636 to 651, 652 to 667, 668 to 682, 707 to 721, 722 to 736, 737 to 751, 764 to 778, 779 to 793, and 839 to 853. Furthermore, in strain Σ1278b Flo11p, there are repeats of 14 identical copies of the sequence APTPSSSTTESSSAP in RII at positions 253 to 267, 268 to 282, 295 to 309, 310 to 324, 361 to 375, 376 to 390, 442 to 456, 484 to 498, 511 to 525, 577 to 591, 592 to 606, 658 to 672, 700 to 714, and 844 to 858, whereas in *S. cerevisiae* var. *diastaticus* there is a single copy at positions 605 to 619 and in strain S288C there are two copies at positions 527 to 541 and 611 to 625. However, it is also interesting that the potential amyloid-forming sequences are conserved in all three strains: VVSTTV in RIII at positions 1033 to 1038 in strain Σ1278b, ITTFV at positions 1050 to 1055, and VTTAVTTTVV in the C-terminal region at positions 1133 to 1142 in strain Σ1278b (23).

### Expression of Flo11p^Σ1278b^ from *S. cerevisiae* var. *diastaticus*.

**FIG 4:**
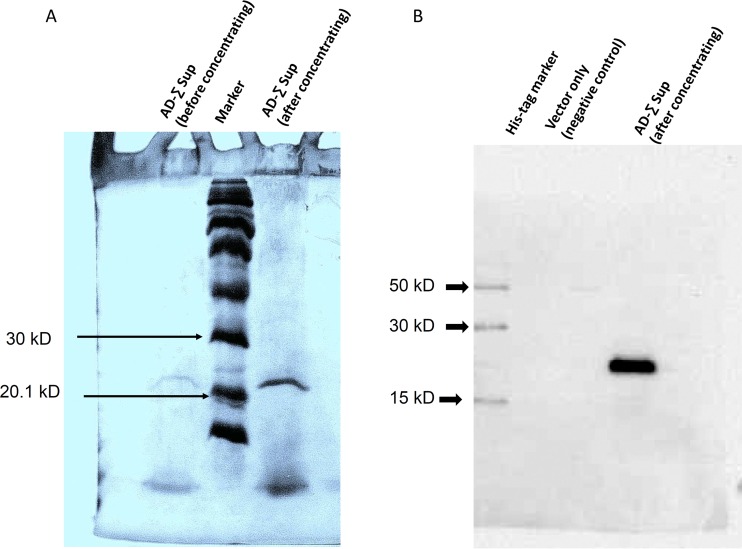
SDS-PAGE and Western blotting of the purified peptide. (A) SDS-PAGE (4% stacking and 15% resolving gels) and Coomassie blue staining of purified supernatant (Sup). Lanes: 1, empty; 2, supernatant from an overnight culture before concentration; 3, Rainbow colored protein high-molecular-weight markers; 4, supernatant from an overnight culture after concentration. (B) Western blot assay probed with peroxidase-conjugated anti-His antibody. Lanes: 1, His tag marker (HisM); 2, supernatant from vector only (negative control); 3, supernatant from overnight culture after concentration.

The strain Σ1278b Flo11p AD (designated AD-Σ) was expressed from *S. cerevisiae* var. *diastaticus flo11Δ* mutant cells. SDS-PAGE was performed from a concentrated culture supernatant. Cells expressing the AD showed an intense band with a molecular mass of ~23 kDa, as expected for the purified protein (Fig. 4A). Immunodetection with an antibody to His_6_ revealed a strong signal corresponding to the ~23-kDa molecular mass of the concentrated product (Fig. 4B). As expected, no such signal was detected in the supernatant from cells harboring the empty vector Yeplac181PGK1p.

### Beads coated with AD-Σ bound preferentially to strain Σ1278b cells.

**FIG 5:**
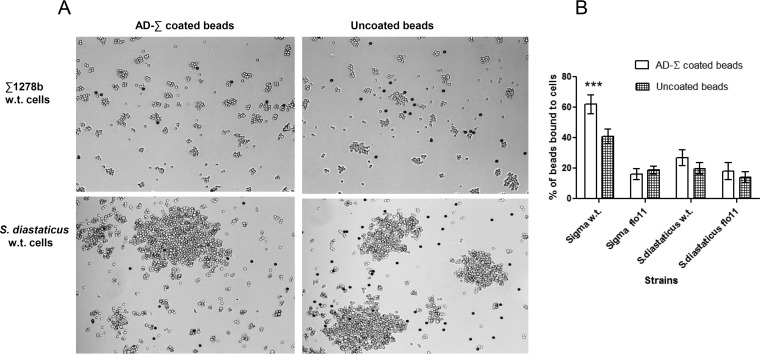
Adhesion to beads coated with the strain Σ1278b Flo11p AD. (A) Beads coated with the strain Σ1278b Flo11p AD (AD-Σ) were allowed to adhere to cells of *S. cerevisiae* var. *diastaticus* and strain Σ1278b, mixed, and photographed. Dark particles are protein-coated beads. Beads were counted and separated into two categories, those bound to yeast cells and those not bound to yeast cells, with a light microscope with a 40× objective (Leica Microsystems, Inc., Allendale, NJ). Strain Σ1278b AD-coated beads bound specifically to Flo11p-expressing cells of strain Σ1278b and poorly to those of *S. cerevisiae* var. *diastaticus*. Uncoated beads were used to control for nonspecific binding. (B) Quantification of the ability of beads coated with the Flo11p amino-terminal domain of strain Σ1278b (AD-Σ) to adhere to different yeast strains. Uncoated beads represent nonspecific binding. Unpaired *t* tests were used, and data are expressed as the mean ± the standard deviation (*n* = ≥3); asterisks denote statistically significant differences (***, *P* < 0.001). Each column represents at least three independent experiments, with at least 200 beads counted for each. w.t., wild type.

Strain Σ1278b AD-coated beads were aggregated with strain Σ1278b or *S. cerevisiae* var. *diastaticus* cells and then vortex mixed, and the number of beads that adhered to the cells was quantified. A majority of the beads were bound to strain Σ1278b cells, whereas most uncoated beads were not bound to cells (Fig. 5). Binding was dependent on *FLO11* expression, because *flo11*Δ mutant cells bound poorly. In contrast, there was no significant difference in the binding of uncoated beads or strain Σ1278b AD-coated beads to *S. cerevisiae* var. *diastaticus* FLO11 or *flo11*Δ mutant cells (Fig. 5).

### Beads coated with AD-S bound preferentially to *S. cerevisiae* var. *diastaticus.*

Sequences encoding the amino-terminal domain of Flo11p from the S288C strain background (AD-S) were also amplified and cloned into the vector. This AD-S sequence is identical to that from *S. cerevisiae* var. *diastaticus* (Fig. 3)*.* Since it has been established that Flo11p is a glycoprotein (16), we also investigated the possibility that different glycosylation patterns of different strains of yeast may yield Flo11p proteins with different properties. Therefore, the plasmid with the cloned AD-S domain was used to transform cells of three different strain backgrounds, namely, *S. cerevisiae* var. *diastaticus*, Σ1278b, and S288C.

**FIG 6:**
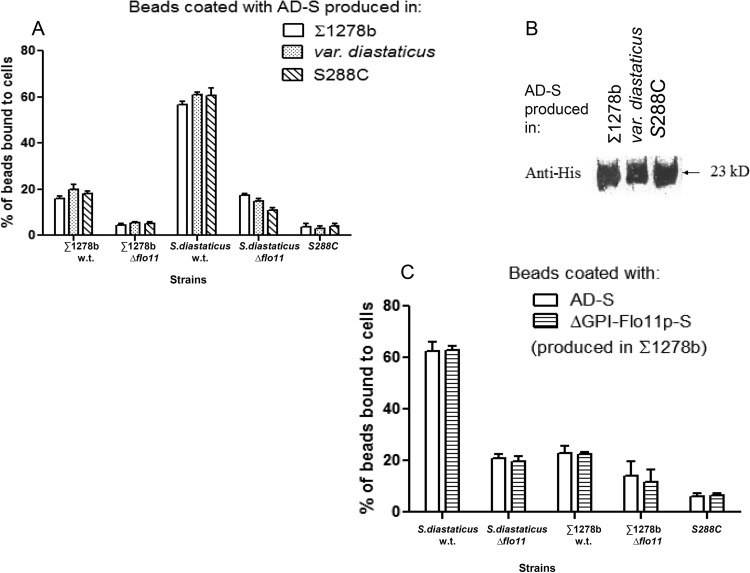
Characteristics of ADs from *S. cerevisiae* S288C. (A) Quantitative bead adhesion assay data show that Dynal beads coated with the same amount of secreted AD-S protein produced by Σ1278b strain L5478, *S. cerevisiae* var. *diastaticus* strain YIY345, or S288C strain BY4741 bound to different strains of yeast. Each column represents the mean ± the standard deviation of three independent experiments, with at least 200 beads counted per experiment. (B) Sequences encoding the amino-terminal domain (AD-S) of Flo11 from the S288C strain background were used to transform cells of three different backgrounds, namely, strain Σ1278b, *S. cerevisiae* var. *diastaticus*, and strain S288C. Western blot assays of culture supernatant with anti-His_6_ antibodies revealed a peptide of approximately 23 kDa from all three plasmid-bearing strains. (C) Quantitative bead adhesion assay data show that Dynal beads coated with the same number of moles of either secreted AD-S or ΔGPI-Flo11p-S produced in Σ1278b strain L5478 bound to *S. cerevisiae* var. *diastaticus* cells. Each column shows the mean ± the standard deviation of three independent experiments, with at least 200 beads counted per experiment. w.t., wild type.

The culture supernatants from all three strains were concentrated, and equal amounts of peptides were used to coat Dynal M-450 tosyl-activated microscopic beads (Dynal Biotech, Lake Success, NJ) in accordance with the manufacturer’s instructions. The coated beads were mixed with cells of the different yeast strains, and bead-to-cell adhesion was observed microscopically. The strain of yeast from which the protein was derived did not significantly affect the binding properties of the beads (Fig. 6A). Beads coated with AD-S bound very effectively to cells of strain *S. cerevisiae* var. *diastaticus*, which is a highly flocculent strain, but much less effectively to cells of strain Σ1278b, which flocculates poorly (Fig. 6A). Binding was dependent on the presence of Flo11p on the cells, since strains with deletions of *FLO11* did not bind the coated beads. Virtually no binding to strain S288C, which does not express *FLO11*, was detected. Control reactions with uncoated beads also showed no significant binding (data not shown). These data are similar to those previously obtained with beads coated with the entire Flo11p protein (16).

Western blot assays of culture supernatant with anti-His_6_ antibodies revealed a peptide of ~23 kDa from all three plasmid-bearing strains (Fig. 6B). This molecular mass is very close to that predicted from the amino acid sequence of this domain. Therefore, if this domain is posttranslationally modified, the modification is insufficient to cause a shift in gel mobility.

### Flo11p AD-S is as effective as ΔGPI-Flo11p-S in causing adhesion to cells.

We compared the activity of AD-S with that of corresponding intact ΔGPI-Flo11p-S, which lacks only the GPI addition signal. Both versions of the protein were produced in strain Σ1278b. Equimolar amounts of the concentrated AD-S and ΔGPI-Flo11p^S288C^ proteins were used to coat beads, the beads were mixed with different yeast strains, and the fraction of beads bound to cells was determined. Figure 6C shows that AD-S-coated beads bound to flocculent *S. diastaticus* cells almost as well as beads coated with equimolar amounts of ΔGPI-Flo11p-S. Strains of the Σ1278b background, which did not flocculate, exhibited much less binding to beads coated with either AD-S or ΔGPI-Flo11p-S (Fig. 6C, middle). However, AD-S-coated beads bound slightly more to strain Σ1278b cells than did ΔGPI-Flo11p-S-coated beads. Strain S288C cells, which do not express *FLO11*, did not bind either type of bead. Therefore, the amino-terminal domain mediates adhesion activity similar to that of the full-length Flo11p protein.

## DISCUSSION

Our data help to resolve a long-standing paradox about the Flo11p adhesin of *S. cerevisiae*: that Flo11p mediates strong flocculation in some strains and its flocculation activity is weak in others, but it has strong activity in the formation of fungal mats and in agar invasion. The differences in flocculation activity result from allelic differences in the sequence of Flo11p, rather than from differences in gene expression levels or posttranslational modification.

Flo11p from either *S. cerevisiae* strain Σ1278b or S288C mediated the formation of cellular aggregates. However, the aggregates of *S. cerevisiae* var. *diastaticus* were much larger (Fig. 5). In contrast, *flo11Δ* mutant cells and strain S288C cells failed to bind to beads coated with either AD, consistent with the requirement for homotypic binding (7, 16). Therefore, there was homotypic binding in strain Σ1278b, as well as in *S. cerevisiae* var. *diastaticus* Flo11p, but in strain Σ1278b, it is not strong enough to cause macroscopic flocculation (16).

Flo11p from nonflocculent strain Σ1278b is composed of 1,360 amino acid residues, compared to 1,361 in alleles from *S. cerevisiae* var. *diastaticus* and strain S288C. Nevertheless, the sequence alignment shows that Flo11p sequences of nonflocculent strain Σ1278b exhibited substantial variations in comparison to *S. cerevisiae* var. *diastaticus* and strain S288C (Fig. 3). The sequence of the strain Σ1278b Flo11p AD has 15 amino acids inserted at residue 115 in the AD (NTDWIDNPLVSRCDE), as well as several amino acid substitutions. The insertion is between β-strands 5 and 6 and is close to two Trp residues that mediate essential hydrophobic-effect interactions between ADs. Therefore, the insertion would be expected to reduce Flo11p homotypic interactions and decrease flocculation activity in strain Σ1278b, either because the insertion alters the conformation in this critical region or because it sterically masks part of the binding site (6, 7). It is less likely but possible that amino acid substitutions alter the domain conformation. Three of these substitutions are conservative (D54N, Q176E, and S179C), and two are nonconservative (W94S in strand β-4 and D189Y in strand β-10). Thus, the 15-residue insertion in strain Σ1278b is likely to prevent the strong homotypic interactions characteristic of flocculation.

The Flo11p ADs showed allele-specific binding specificity, as expected if the sequence differences in the ADs are important. Beads coated with AD purified from strain Σ1278b or S288C (identical to the sequence of *S. cerevisiae* var. *diastaticus*) showed preferential binding to cells expressing the homologous adhesin (Fig. 5 and 6). In addition, the Flo11p AD from strain S288C showed similar binding specificity when expressed from any of three strains, *S. cerevisiae* var. *diastaticus*, Σ1278b, or S288C. This result implies that differences in binding specificity and flocculation induction are not due to strain-specific posttranslational processing of Flo11p.

Binding activity and specificity were also similar for beads coated with the AD only or with ΔGPI-Flo11p-S, which lacks only the C-terminal 30 of the 1,360 amino acids (Fig. 6C). This protein includes extensively glycosylated serine/threonine-rich regions that form stiff rod-like structures that facilitate extracellular exposure of the amino-terminal domain (24). In *Candida albicans* Als5p, as well as in Flo1p and in *S. cerevisiae* var. *diastaticus* Flo11p, this region folds into multiple small compact domains that are unfolded by extension in the atomic force microscope (23, 26, 38, 39). Indeed, in the *C. albicans* adhesin Als5p, the repeats interact through a hydrophobic effect and stabilize binding to fibronectin (28). In Flo1p, the results are consistent with homotypic binding between the repeats, and variations in repeat numbers cause phenotypic variations in flocculation (23, 25, 26). The Flo11p sequences show a high degree of sequence similarity between the Flo11p repeats in the three strains, but at least 15 insertion/deletion events are evident between the strain Σ1278b allele and the *S. cerevisiae* var. *diastaticus* allele. There have been two insertion/deletion events in this region between *S. cerevisiae* var. *diastaticus* and strain S288C. Because the ADs are sufficient to confer flocculation specificity (Fig. 5 and 6), changes in the tandem repeats appear to cause secondary or minor differences in Flo11p flocculation activity.

The strains also showed differences in the expression levels of Flo11p and in the contribution of Flo11p to surface hydrophobicity. *FLO11* transcriptional regulation depends on the growth phase; there was significantly higher expression in the stationary phase of each strain than in the log phase (Fig. 1A). These data are consistent with the previous findings that *FLO11-*dependent phenotypes are more prominent during glucose starvation (8, 14). Interestingly, *FLO11* mRNA expression in strain Σ1278b was significantly higher than that in *S. cerevisiae* var. *diastaticus* (Fig. 1B). Despite its lower transcript level, Flo11p in *S. cerevisiae* var. *diastaticus* is sufficient to ensure a high level of flocculation.

In strain Σ1278b, Flo11p contributed about 40% of the surface hydrophobicity. *S. cerevisiae* var. *diastaticus* was substantially more hydrophobic, and partitioning to the nonpolar phase was three times as great. Deletion of *FLO1* decreased nonpolar partitioning by about 10% in each strain, but the difference was a greater fraction of the partition in strain Σ1278b, because of its lower basal hydrophobicity. Indeed, hydrophobicity analyses of Flo11p sequences of strain Σ1278b and *S. cerevisiae* var. *diastaticus* show similar hydrophobicity profiles for both alleles (http://web.expasy.org/protscale/) (27, 29). Therefore, neither transcription level nor surface hydrophobicity accounted for the activity differences in the *FLO11* products.

In summary, our data support the idea that strain-specific differences in the level of flocculation are due to sequence differences in the ADs. The sequence of the AD of nonflocculent strain Σ1278b varies significantly from that of highly flocculent *S. cerevisiae* var. *diastaticus*, and each allele showed strain-specific homotypic binding. These results were similar, regardless of the strain used to produce the Flo11 AD, a result demonstrating that strain-specific differences in the expression level or posttranslational modifications have little effect. In addition, allelic differences outside the ADs had little effect on binding specificity. Thus, the data are consistent with the idea that sequence differences in the AD (including a 15-amino-acid insertion near the binding surface in a nonflocculent strain) determine the flocculation activity of Flo11p.

## MATERIALS AND METHODS

### Real-time qRT-PCR analysis for assessment of *FLO11* mRNA expression.

The yeast strains listed in Table 2 were grown overnight in yeast extract-peptone-dextrose medium at 30°C to an optical density at 600 nm (OD_600_) of ~0.8 for log phase or to an OD_600_ of ~2.8 for stationary phase. Total RNA was isolated by the hot acidic-phenol method (30). Each sample was treated with RNAsecure reagent (Ambion, Inc., Austin, TX). Extracted total RNA was further treated with RNase-free DNase (Qiagen, Inc., Valencia, CA) to get rid of any DNA contamination and purified with the RNeasy kit in accordance with the manufacturer’s instructions (Qiagen, Inc., Valencia, CA). Quantitation of RNA was performed by measuring the *A*_280_ with RNase-free water as the blank. Bio-Rad MyIQ single-color real-time PCR detection systems were used with the iScript kit (Bio-Rad Laboratories, Hercules, CA) and real-time PCR amplification with the same amount of RNA for all samples. Expression of *FLO11* mRNA was normalized to the relative expression value of the housekeeping gene *PDA1* (31) in each sample. The qRT-PCR cycling conditions were 50°C for 2 min, 95°C for 10 min, and 40 cycles of 95°C for 15 s and 59°C for 1 min. The primers used for amplification of *FLO11* mRNA were Forward FLO11 (5′ TCGCTTATTTGGTCCTTTCG 3′) and Reverse FLO11 (5′ AAGTTGGGACAGCCATTAAC 3′), and those used for the housekeeping gene *PDA1* were Forward PDA1 (5′ GGAATTTGCCCGTCGTGTT 3′) and Reverse PDA1 (5′ GCGGCGGTACCCATACC 3′).

**TABLE 2:** Yeast strains and plasmids used in this study

Strain name	Background	Genotype or description	Reference or source
Strains			
YIY345	*S. cerevisiae* var. *diastaticus*	*MAT***a** *ura3 leu2-3*,*112 his4*	35
YIY345flo-1	*S. cerevisiae* var. *diastaticus*	*MAT***a** *ura3 leu2-3*,*112 his4 flo11*::*URA3*	17
L5487	Σ1278b	*MAT*α *leu2*::*hisG ura3-52*	36
L5487flo11Δ	Σ1278b	*MAT*α *leu2*::*hisG ura3-52 flo11*::*URA3*	14
L5486	Σ1278b	MAT**a** *leu2*::*hisG ura3-52*	36
FY2	S288C	*MAT*α *ura3-52*, *GAL2^+^*	37
BY4741	S288C	MAT**a** *his3*Δ*1 leu2*Δ*0 met15*Δ*0 ura3*Δ*0*	SGD^a^
Plasmids			
pFLO11		YEplac181-PGK1p-FLO11-S-GPIΔ	16
pAD-FLO11-Σ		YEplac181-PGK1p-FLO11-Σ-AD	This work,^b^ 16
pAD-FLO11-S		YEplac181-PGK1p-FLO11-S-AD	This work,^b^ 16

a SGD, *Saccharomyces* Genome Database.

b Derived from pFLO11.

### Hydrophobicity assay.

Yeast cell surface hydrophobicity was assessed by an aqueous-hydrocarbon biphasic hydrophobicity assay adapted from a method previously described (15, 32). Briefly, cells were grown in synthetic complete (SC) medium to an OD_600_ of ~3.0; cells were then resuspended to an OD_600_ of ~0.8. A 1.2-ml culture sample was added to a 13- by 100-mm borosilicate glass tube. A 600-µl volume of octane was used to overlay the cell suspension, which was then vortexed for 3 min. The biphasic layers were allowed to separate, and then the OD_600_ of the aqueous layer was measured. The relative difference between the OD_600_ of the aqueous phase before and after the addition of octane was used to determine the percentage of hydrophobicity.

### PCR amplification of the sequencing product.

Yeast genomic DNA from *S. cerevisiae* var. *diastaticus* strain YIY345 (generously provided by I. Yamashita; Table 2) was extracted from the overnight culture. PCR primers were designed on the basis of the strain S288C *FLO11* DNA sequence and amplified for DNA sequencing. Because the serine/threonine-rich region was too repetitive to permit the design of a unique specific primer, sequencing was done by amplifying 1.8 kb of the repeat region with forward and reverse primers designed on the basis of the sequences upstream and downstream of the repeat. PCR was carried out at 94°C for 4 min; 94°C for 1 min, 58°C for 1 min 30 s, and 72°C for 3 min for 25 cycles; and a final extension at 72°C for 10 min. The amplified product was digested with restriction enzymes MmeI and SfcI (New England Biolabs, MA). The gel-purified digested product was ligated with a linker having a universal primer site at the 5′ end (*t*7, 5′ TAATACGACTCACTATAGGG 3′; GENEWIZ, North Brunswick, NJ). The resulting product was purified with the QIAquick PCR purification kit (Qiagen, Valencia, CA) to eliminate linkers. The purified ligated product was then amplified by PCR with specific primers homologous to the linker sequence and the genomic DNA sequence. The amplified PCR product was further purified with the QIAquick PCR purification kit (Qiagen, Valencia, CA) in accordance with the manufacturer’s protocol. Sequencing was done with primers specifically designed by the GENEWIZ sequencing facility (North Brunswick, NJ).

### Plasmid construction.

Plasmid YEplac181-PGK1p-MUC1-GPIΔ (16, 33), having the *FLO11* sequence from the strain S288C background, was initially digested with restriction enzymes EcoRI and SalI (New England Biolabs, MA) to cut out the *FLO11* gene. Purification of the empty vector Yeplac181PGK1p was performed with the Qiaex II gel extraction kit (Qiagen, Inc., Valencia, CA) in accordance with the manufacturer’s recommendation. The insert DNA, having the AD from the strain Σ1278b (encoding AD-Σ) background (generously provided by members of the laboratory of Gerald Fink; Table 2) without a GPI anchor, was created. Primers 5′ CGAGAATTCATGCAAAGACCATTTCCATTCGCTTATTTGGTCCTTTCGCTTC 3′ and 5′ ATCGTCGACTTAGTGGTGATGGTGATGATGACAATTGTTGTCACAATCTATGTTCC 3′, having EcoRI and SalI restriction sites and a His_6_ tag at the 5′ end, were used to amplify the AD of strain Σ1278b. The PCR amplification cycles were programmed as follows: 94°C for 4 min (denaturation); 94°C for 1 min, 50°C for 1 min 30 s, and 72°C for 3 min for 25 cycles; and a final extension at 72°C for 10 min.

For pADFLO11-GPIΔ for the AD-S, the BamHI site in the multicloning region was deleted, leaving the BamHI site at the end of the signal sequence in *FLO11* as a unique restriction site in the resulting construct, p*FLO11*-GPIΔ-Bamless. p*FLO11*-GPIΔ-Bamless was digested with BamHI and PstI to release most of the coding sequence of *FLO11* (including nucleic acids 88 to 3993). The remaining 7.4-kb plasmid fragment containing the *FLO11* signal sequence and a His_6_ tag was gel purified with a gel extraction kit (Qiagen Inc., Valencia, CA) and served as the vector for DNA encoding the AD of Flo11 (pAD). The AD of Flo11p from *S. cerevisiae* var. *diastaticus* was amplified by PCR with a forward primer with a BamHI site and a reverse primer with a PstI site. The forward primer was 5′ ATTCCT**GGATCC**TCCGAAGGAACTAGCTG 3′, and the reverse primer was 5′ ATCTCA**CTGCAG**ACTTCGTACCGCCACAATTATTGT 3′ (the restriction sites are in bold).

After amplification, to create padflo11-gpiΔ (for AD-Σ), the amplified product was first purified with the QIAquick PCR purification kit (Qiagen, Inc., Valencia, CA). The PCR product was then digested with restriction enzymes EcoRI and SalI (New England Biolabs, MA) and ligated into the cleaved EcoRI and SalI sites of the vector. For padflo11-gpiΔ (for AD-S), after purification, the amplified PCR product was digested with restriction enzymes BamHI and PstI (New England Biolabs, MA) and ligated into the cleaved BamHI and PstI sites of the vector. Both of the plasmids were transformed into *E. coli* Library Efficiency DH5α competent cells (Invitrogen Life Technologies, Carlsbad, CA) in accordance with the manufacturer’s instructions.

### Expression and purification.

*S. cerevisiae* var. *diastaticus* and strains Σ1278b and S288c were transformed with pADFLO11-GPIΔ to encode ad-Σ, AD-*S*.*d*., or AD-S and then grown in SC medium without leucine and with two times the concentration of amino acids in SC medium for growth enhancement. Incubation at 30°C was carried out until stationary phase, i.e., an OD_600_ of approximately 4.0; when supernatants were collected. Secreted proteins in the supernatants were concentrated with Amicon Ultra-4 filter devices with a 10,000 molecular weight cutoff (catalog no. UFC801024; Millipore, Corp., Billerica, MA). Further concentration of the concentrated proteins was performed with Amicon Ultra-0.5 filters with a 10,000 molecular weight cutoff. The total protein in the concentrated sample was quantified by the Bradford method (Coomassie Plus Protein Assay Reagent; Pierce Biotechnology, Perbio, Rockford, IL). SDS-PAGE of the concentrated product was performed along with culture supernatant of *S. cerevisiae* var. *diastaticus flo11Δ* mutant cells having Yeplac181PGK1p (vector only) as a negative control. Protein gel electrophoresis was performed with 4 to 15% SDS-polyacrylamide gel, and samples were run at 80 V for 120 min. SDS-PAGE gels were stained for 1 h or overnight in 100 ml of Coomassie blue staining solution with gentle rocking.

### Immunoblotting.

Immunoblotting of proteins blotted onto polyvinylidene difluoride membrane (Immobilon Millipore P, Millipore Corp., Billerica, MA) was performed with a mouse IgG_2b_ primary monoclonal antibody specific for a consecutive sequence of six histidine residues (Invitrogen Life Technologies, Carlsbad, CA) diluted 1:5,000 in 10 ml of blocking buffer for 3 h at room temperature or with a mouse immunoglobulin G (IgG) primary monoclonal antibody specific for a consecutive sequence of five histidine residues (Penta-His; Qiagen, Inc., Valencia, CA) diluted 1:2,000 in Tris-buffered saline with Tween 20 plus 5% milk for 1 h. Membranes were then exposed to a horseradish peroxidase-conjugated anti-mouse IgG secondary antibody for 15 min to 1 h at room temperature with gentle agitation. Detection of the His-tagged protein was done with ECL chemiluminescence assay Western detection reagents (Amersham Pharmacia Biotech, Inc., Piscataway, NJ) for 30 s in accordance with the manufacturer’s instructions.

### Preparation of protein-coated beads.

Concentrated Flo11p protein was used to coat *p*-toluenesulfonyl chloride-activated, tosyl-activated Dynal M-450 beads (Dynal Biotech, Inc., Lake Success, NY) by the method of Douglas et al. (16). In a 0.5-ml tube, 10^7^ beads were washed and resuspended for 2 min in 100 µl of buffer A (0.1 M sodium phosphate, pH 7.4). A 5-µg sample of the Flo11p AD was added, and the combination was mixed by brief vortexing. Beads were then incubated for ~36 h at 30°C with slow tilt rotation. Beads were collected with a magnetic stand for 2 min, and the supernatants were pipetted off to a separate tube (the OD_595_ of the supernatant was measured again). After the beads were washed twice for 5 min in phosphate-buffered saline (PBS), pH 7.4, they were collected with a magnetic stand and the supernatants were discarded. Washing was then performed with 0.2 M Tris, pH 8.5, with 1.5% nonfat dry milk or fetuin as a blocking agent. Incubation was then done at 30°C for 1 h, followed by ~36 h at 4°C with slow tilt rotation. After the beads were collected with a magnetic stand, washing was performed in PBS, pH 7.4, for 5 min at 4°C. Beads coated with nonfat dry milk or fetuin only were included as a control in each experiment.

### Bead-based adhesion assay.

A bead-based adhesion assay was performed by a modification of the method of Gaur et al. (34). Cells were grown overnight to a density of ~2 × 10^8^/ml. Aliquots of ~1 × 10^8^ cells were washed twice in deflocculation buffer (20 mM sodium citrate, 5 mM EDTA, pH 4.0), the cells were resuspended in deflocculation buffer containing 20 mM calcium chloride to promote adhesion (8), 1 × 10^6^ coated beads (2.5 µl) were added to a total volume of 1 ml, and then the cells plus protein-coated beads were incubated in SC medium with 0.1% glucose for 1 h 30 min at 30°C with slow tilt rotation. (The assay was carried out in this medium because strain Σ1278b requires glucose starvation for maximum Flo11-dependent adhesion.) (15). After the reaction mixture was vortexed vigorously for ~20 s, wet mounts on glass slides were prepared immediately for microscopic viewing. Beads were counted and separated into two categories, beads bound to yeast cells and beads not bound to yeast cells, with a light microscope with a 40× objective (Leica Microsystems, Inc., Allendale, NJ). For each category, values were calculated and are presented as percentages based on the total number of beads counted with respect to that category (16).
